# Comprehensive Surgical Management of Massive Oesophageal Hiatal Hernia in Squirrel Monkeys (*Saimiri sciureus*)

**DOI:** 10.1002/vms3.70404

**Published:** 2025-05-19

**Authors:** Siyuan Li, Zhenwen Zhang, Md. F. Kulyar, Shah Nawaz, Jiakui Li

**Affiliations:** ^1^ College of Veterinary Medicine Huazhong Agricultural University Wuhan China; ^2^ College of Veterinary Medicine Nanjing Agricultural University Nanjing China

**Keywords:** gastrointestinal complications, massive hiatal hernia, *Saimiri sciureus*, squirrel monkeys, surgical management

## Abstract

**Background:**

Literature remains scant regarding the subset termed ‘massive hiatal hernias’. This case report delineates the surgical management and subsequent outcomes of two squirrel monkeys diagnosed with massive hiatal hernias.

**Objectives:**

This case report delineates the surgical management and subsequent outcomes of two squirrel monkeys diagnosed with massive hiatal hernias, offering insights into the feasibility and criticality of timely interventions, underscored by postoperative care and dietary management.

**Methods:**

The radiographic diagnosis is suggestive of a massive hiatal hernia, and this clinical suspicion was definitively corroborated during the subsequent surgical intervention. Left gastric fixation was used to reposition the herniated organs and repair and reinforce the hiatus.

**Results:**

One subject died of unforeseeable complications 3 days after post‐discharge. The other subject's hospitalization was extended to 40 days to ensure optimal recovery. Upon being discharged, the monkey's condition remained stable.

**Conclusions:**

This case suggested achieving long‐term postoperative survival of a massive oesophageal hiatal hernia. Surgical management hinges not merely on the surgery but on rigorous postoperative care coupled with stringent dietary regulation.

## Graphical abstract

Radiograph shows displacement of soft tissue from the abdominal region. Abdominal surgery was conducted to reposition the organ, repair the hernia and gastropexy. Case underscores the surgical intervention and the indispensable nature of postoperative care and dietary management in improving the prognosis for animals with hiatal hernias.

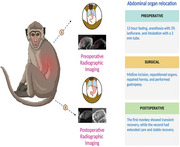

## 1 Introduction

A hiatal hernia characterizes the anomalous protrusion of abdominal organs into the thoracic cavity via the oesophageal hiatus of the diaphragm, emerging as a prevalent disorder within the gastrointestinal domain (Rochefort and Wee 2018; Sfara and Dumitrascu 2019). Accumulating evidence has identified instances of hiatal hernias in both humans and various animal species. However, literature remains scant regarding the subset termed ‘massive hiatal hernias’ (DeSandre‐Robinson et al. 2011; Greene et al. 2015; Hettlich et al. 2010). While the precise delineation of ‘massive hiatal hernia’ remains somewhat nebulous, current clinical frameworks predominantly identify such hernias as those possessing a diameter exceeding 5 cm. Another prevalent characterization denotes a massive hiatal hernia where 30%–50% of the stomach's volume abnormally transitions into the mediastinal region (Geha et al. 2000; Oelschlager et al. 2006). Modern taxonomies divide hiatal hernias into four discernible types grounded on the relative positioning of the gastroesophageal junction (Sfara and Dumitrascu 2019; Siegal et al. 2017). However, the precise aetiological factors and pathophysiological underpinnings, particularly those associated with potential anomalies of the lower oesophageal sphincter, remain a subject of ongoing investigation across both human and animal research domains (Sivacolundhu et al. 2002).

## 2 Case Presentation

**FIGURE 1:**
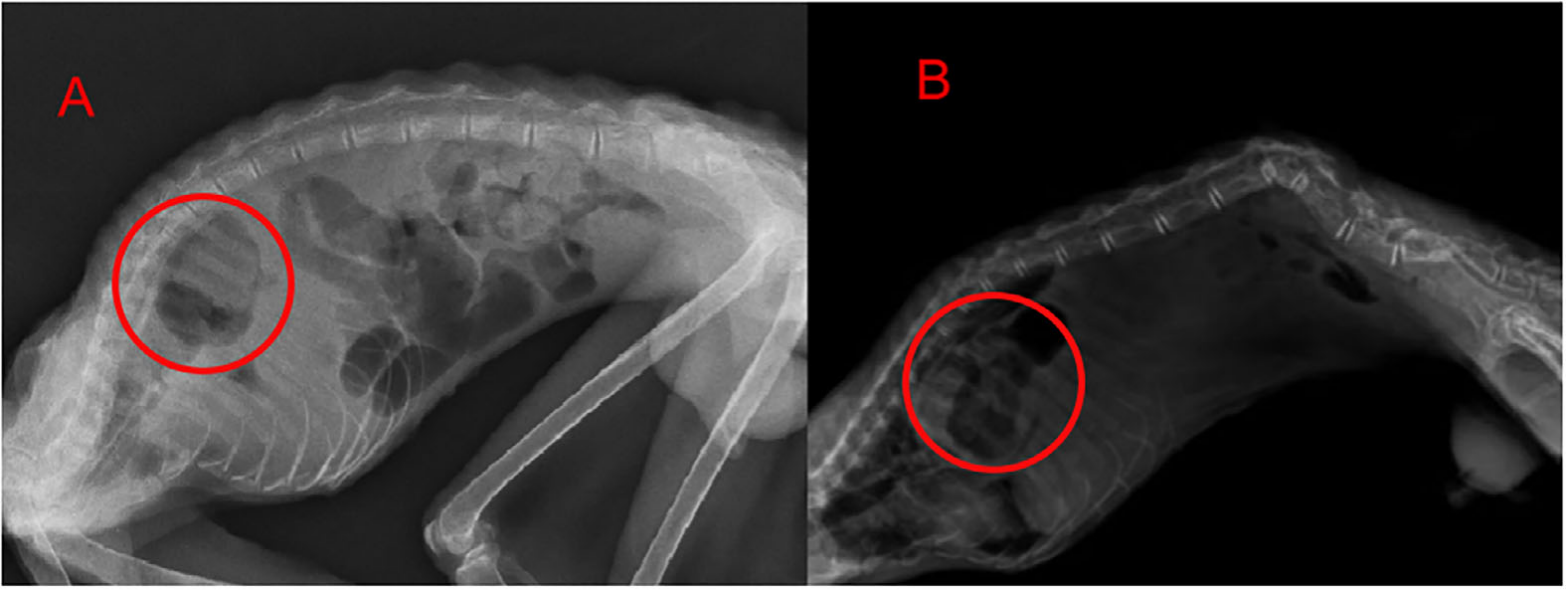
The detailed preoperative anteroposterior radiographs. (A, B) The images illustrate the aberrant transition of a significant portion of abdominal contents into the thoracic cavity, consistent with the features of a massive hiatal hernia cases.

Two juvenile male squirrel monkeys, approximately 3 years of age and hailing from a local wildlife reserve, were referred to our veterinary medical facility. The primary presenting complaints were marked emaciation and overt behavioural depression. The subject was admitted with no prior medical records, as they originated from a wildlife reserve with limited health surveillance. While traumatic aetiology was considered, no physical or radiographic evidence of injury (e.g. fractures, soft tissue damage) was identified during preoperative assessment. Upon clinical evaluation, both subjects were in a severe state of malnutrition, weighing in at 530 and 430 g, respectively. The physical examination revealed clinical dehydration and tachypnoea, with a notably elevated respiratory rate of 105 breaths/min, heart rate was approximately 150 beats/min. The patient's pre‐operative complete blood count (CBC) and blood chemistry showed some degree of anaemia (RBC 5.26 × 10^12^/L and 4.85 × 10^12^/L, HGB 72 g/L and 68 g/L) and malnutrition (ALB 17.5 g/L 16.3 g/L). Diagnostic imaging via anteroposterior and lateral radiographs unveiled a conspicuous soft tissue mass translocating from the abdominal compartment to the thoracic region via the diaphragmatic hiatus, consistent with a massive hiatal hernia (Figure 1). Additional pre‐operative assessments included CBC, serum biochemistry (e.g. albumin, liver enzymes) and physical examination to evaluate systemic health status. This clinical suspicion of a massive hiatal hernia was definitively corroborated during the subsequent surgical intervention.

Upon reaching a conclusive diagnosis of a massive hiatal hernia, a comprehensive surgical approach was meticulously charted out to address the intricate anatomical challenges posed by this condition in our juvenile squirrel monkey subjects.

**FIGURE 2:**
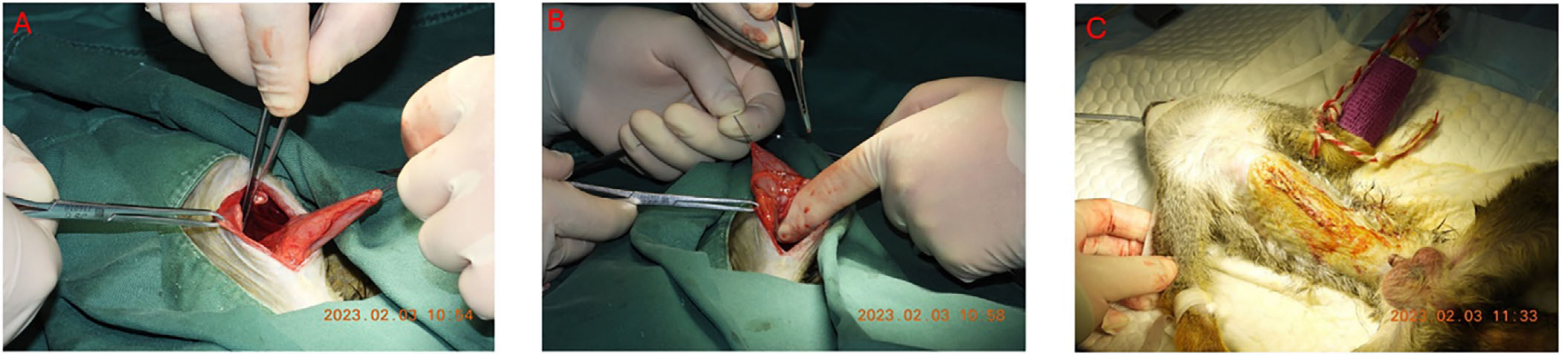
Surgical intervention of hiatal hernia. (A) Soft tissue mass translocating. (B) Hernia repair and gastric fixation to enhance the robustness of our intervention.

The foremost step was to carefully relocate the herniated abdominal contents, predominantly the stomach and oesophagus, back to their native anatomical position within the abdominal cavity. This aimed to alleviate the immediate symptoms and ensured that potential postoperative complications could be pre‐emptively mitigated. After successfully repositioning the herniated organs, attention was directed towards addressing the hiatal defect, which was the root cause of the herniation. The oesophageal hiatus was surgically repaired and reinforced to ensure long‐term stability and minimize the recurrence risk. Considering the inherent risk of gastroesophageal reflux post‐hernia repair, anti‐reflux measures were integrated into the surgical protocol. This entailed creating a barrier to retrograde flow from the stomach back into the oesophagus. Lastly, gastric fixation was performed to enhance the robustness of our intervention further and to tether the stomach securely. This step was executed based on real‐time intra‐operative findings and the surgical team's informed discretion, ensuring a holistic and effective surgical resolution of the hiatal hernia (Figure 2).

To minimize anaesthetic complications, subjects underwent a 12‐h pre‐operative fasting period. A mixture of glucose and rehydration salts was provided for drinking during fasting. About 5% dextrose was administered intravenously at a rate of 10 mL/h starting 1 h before surgery.

Atropine sulphate (0.05 mg/kg, IM) was administered 15 min prior to induction to minimize bronchial secretions and vagal reflexes. Dexmedetomidine hydrochloride (0.02 mg/kg, IM) was injected to achieve α₂‐adrenoceptor‐mediated sedation and analgesia. For anaesthetic induction, 2% isoflurane was administered with non‐rebreathing circuit. Following effective induction, the subjects were intubated with a suitably sized cuffed endotracheal tube (2 mm internal diameter, Mindray WATO EX‐20) and maintained on a 1%–2% isoflurane and 100% inhalational anaesthetic regimen throughout the surgical procedure, as referenced previously (Brady 2000).

All vital parameters were closely observed during the procedure. The heart rate was 152 ± 6 beats/min on average, respiratory rate 100 ± 4 breaths/min and oxygen saturation (SpO₂) > 92% throughout the procedure. The capillary refill time was < 2 s. Blood pressure could not be measured directly because of a lack of equipment, but indirect indicators like mucous membrane colour and capillary refill time indicated stable haemodynamics. Subcutaneous lidocaine 2% (1 mg/kg) was injected along the planned midline incision site before skin incision.

**FIGURE 3:**
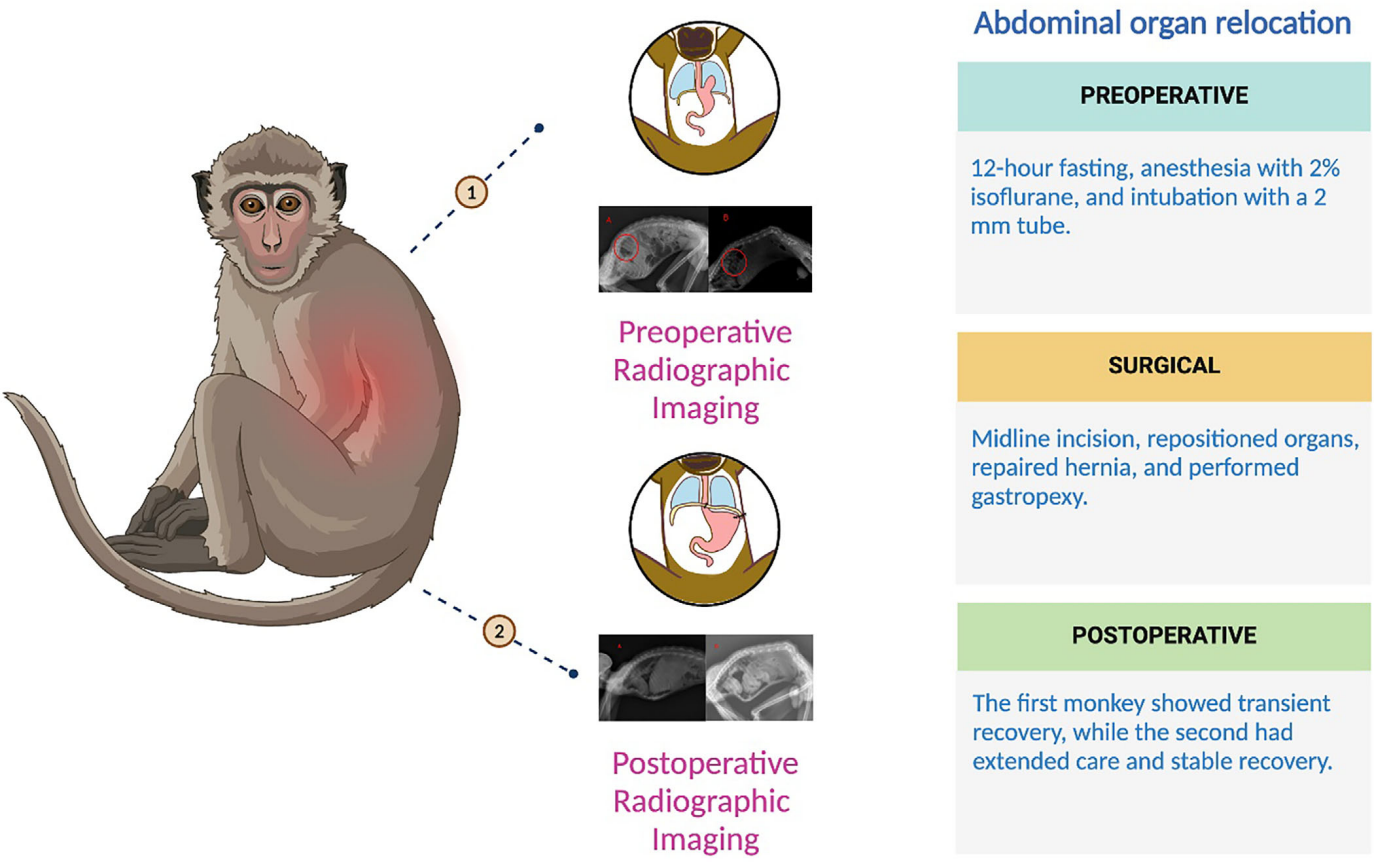
Comprehensive surgical management of a massive oesophageal hiatal hernia in squirrel monkeys (*Saimiri sciureus*). (1) Intraoperative view showing the initial abdominal opening, providing access to the hernia site. (2) Exposure of the massive oesophageal hiatal hernia, demonstrating the extent of the herniation. Postoperative measures are given in text.

**FIGURE 4:**
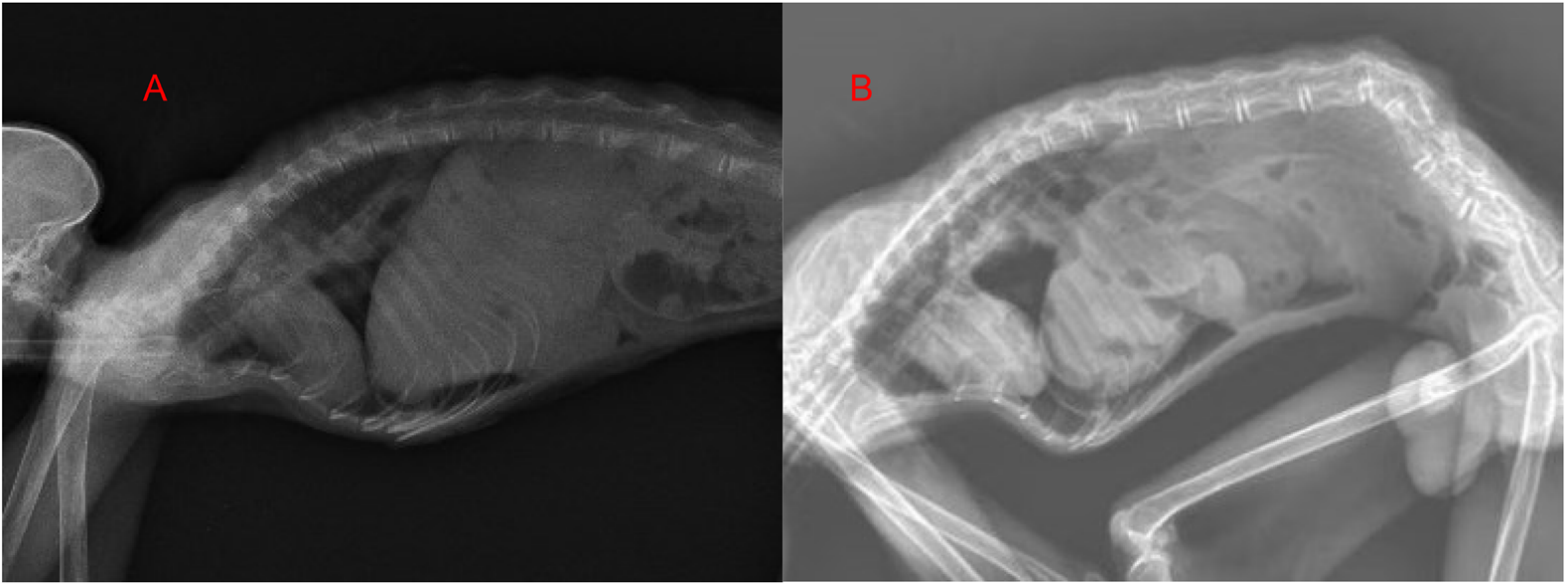
Postoperative radiographic imaging: This set of x‐ray films, captured after the surgical intervention. (A, B) The radiographs provide a detailed visualization of the thoracic and abdominal regions, showcasing the repositioned abdominal organs and the repair site at the oesophageal hiatus.

An abdominal midline incision was performed, followed by careful dissection of the subcutaneous adipose tissue layer. On incising through the abdominal musculature and accessing the peritoneal cavity, it was discerned that approximately 50% of the stomach and oesophagus had aberrantly translocated into the thoracic compartment via the oesophageal hiatus. Notably, there were no signs of adhesions, tissue strangulation or impaired blood perfusion to the herniated contents. Furthermore, no subcutaneous haemorrhagic manifestations or focal tissue necrosis were identified. The hernial sac was meticulously addressed using simple interrupted sutures employing silk, as delineated by Al‐Sobayil and Ahmed (2007). A left‐sided incisional gastropexy was then successfully executed using absorbable suture material to prevent potential recurrence (Figure 3A–C). The x‐ray films captured after the surgical shows showcase the repositioned abdominal organs (Figure 4). IV fluids were continued for 2 h post‐surgery at the same rate (10 mL/h). Ad libitum oral rehydration was achieved through administration of a 5% dextrose solution compounded with oral rehydration salts (ORS), superseding conventional free water intake.

In order to prevent infection, ceftriaxone sodium (30 mg/kg, IM) was administered every 24 h for 72 h post‐operatively. Meloxicam (0.2 mg/kg PO q24h) was initiated and continued for 72 h post‐operatively. Pain assessments were performed every 6 h for 3 days. Butorphanol (0.02 mg/kg IM q6h) if nociceptive reflexes (e.g. tachycardia > 20% baseline, spontaneous movement) were observed. However, the intravenous indwelling needle, which was initially placed for the infusion of glucose and sodium chloride injection, had to be removed due to the patient's lack of cooperation one day after the operation, resulting in the discontinuation of the subsequent fluid replacement.

The first monkey exhibited a positive post‐operative trajectory initially. Within 2 days following the surgical procedure, regular bowel movements were re‐established. Over the subsequent days, the monkey's dietary intake normalized (Videos S1 and S2). By the 10th postoperative day, the clinical team deemed the subject ready for discharge based on its consistent dietary and bowel patterns. However, this recovery was short‐lived. The monkey faced unforeseen complications 3 days post‐discharge, succumbing to circulatory disturbances and pulmonary congestion. Necropsy shows marked gaseous distension of the stomach and small intestine; prominent dehiscence and inflamation along severe pulmonary congestion and oedema, no pleural effusion or pneumothorax; alveolar haemorrhage and neutrophil infiltration, consistent with acute respiratory distress syndrome (ARDS); intact hiatal repair sutures with no signs of dehiscence or inflammation. So, we excluded those causes like surgical failure (e.g. hernia recurrence, suture breakdown) and sepsis. The sudden decline in health was potentially amplified by severe gastrointestinal distension, which may have imposed undue pressure on the thoracic cavity. In stark contrast, given its initially compromised physical health upon admission, the second monkey was slated for surgery 10 days after being hospitalized. It adopted a similar surgical methodology as the first case but incorporated a more extended period of in‐hospital recovery and intensive monitoring. Notably, the monkey began manifesting signs of dyspepsia and pronounced bloating on the seventh day following the surgery. During the observation period, the intake of solid foods was restricted, and the patient was treated with oral glucose and ranitidine. Subsequently, the symptoms resolved within 48 h. Considering these symptoms, the hospitalization was extended to 40 days to ensure optimal recovery. Upon being discharged, the monkey's condition remained stable, and it exhibited signs of recovery, underlining the importance of extended postoperative care and monitoring in such intricate cases.

## 3 Discussion

The precise diagnosis and subsequent classification of a massive hiatal hernia are primarily contingent on evaluating chest radiographs and barium x‐rays. Such assessments, albeit preliminary, should be corroborated by intraoperative findings to ensure accuracy (Fisichella and Patti 2008). Notably, in small animals, the presentation of a hiatal hernia is often skewed towards a medical and surgical emergency, contrasting with its manifestation in humans (Phillips 1987). Surgical intervention remains pivotal, independent of overt clinical manifestations, given the latent, unpredictable complications associated with massive hiatal hernias (Duranceau 2016; Sivacolundhu et al. 2002).

Central to the surgical procedure is the closure of the hiatus. The hiatal canal modulates pressure, even during diaphragmatic relaxation phases. However, the ideal extent of hiatal canal closure remains debatable. Current suggestions advocate for reducing the hiatus's diameter to approximately 1–2 cm, although this is contingent on the specific dimensions of the animal in question. Considering the diminutive stature of the squirrel monkeys, laparoscopic repair was deemed unsuitable. The inherent challenges posed by conditions like volvulus or strangulation further diminish the feasibility of a laparoscopic approach. Moreover, a mid‐surgery transition between surgical modalities inadvertently extends the surgical timeline, thereby amplifying anaesthetic risks, especially in physically compromised small animals.

Left‐side assisted gastropexy emerges as a viable alternative treatment strategy for candidates with advanced age, marked physical instability, or those failing to meet the criteria for hernia sac repair. It is pertinent to note that surgical rectification of a massive hiatal hernia, devoid of complications like incarceration or concurrent organ herniation, is relatively straightforward. While the subjects in this study unfortunately did not achieve long‐term survival, the encouraging postoperative trajectory underscores the relative success of the surgical intervention.

Aetiologically, the hiatal hernia's emergence in these cases can be attributed to congenital developmental anomalies or underlying neurological/neuromuscular disorders. The absence of physical traumatic signs in this case. Nevertheless, the retrospective nature of this study and incomplete clinical history underscore the challenges in definitively excluding trauma in wildlife cases. Future prospective studies with standardized monitoring protocols are warranted to elucidate causative factors. Anatomically, any resultant abdominal distension can have profound ramifications. The diaphragm's relaxation, combined with gastric bloating, exerts pronounced pressure on the cardiovascular and respiratory systems, which, if unchecked, can culminate in acute mortality. Although the operative technique involved, primarily abdominal incision surgery, is not overly intricate and facilitates hernia repair and organ repositioning, laparoscopy remains non‐recommended. Achieving long‐term post‐operative survival hinges not merely on the surgery but significantly on rigorous post‐operative care coupled with stringent dietary regulation. Recognizing and swiftly addressing postoperative bloating as a medical necessity demanding immediate intervention is imperative.

## 4 Conclusions

The surgical management of massive hiatal hernias in squirrel monkeys, underpinned by early diagnosis and intervention, emerges as a viable therapeutic avenue. This case report underscores the potential of surgical intervention and the indispensable nature of postoperative care and dietary management in improving the prognosis for animals with hiatal hernias.

## Author Contributions

**Siyuan Li**: investigation, resources, conceptualization, methodology, validation, visualization, writing – original draft. **Zhenwen Zhang**: investigation, funding acquisition, methodology, resources. **Md. F. Kulyar**: validation, writing, project administration–review and editing. **Shah Nawaz**: writing – review and editing. **Jiakui Li**: project administration, resources, supervision. All the authors read and approved the final manuscript.

## Ethics Statement

The authors confirm that the ethical policies of the journal, as noted on the journal's author guidelines page, have been adhered.

## Consent

Written informed consent was obtained from the animal keeper for publication of this study and accompanying images.

## Conflicts of Interest

The authors declare no conflicts of interest.

### Peer Review

The peer review history for this article is available at https://www.webofscience.com/api/gateway/wos/peer‐review/10.1002/vms3.70404.

## Supporting information



Supporting information

Supporting information

Supporting information

## Data Availability

All data generated or analysed during this study are included in this published article.
