# Supplementary material for: Comprehensive Surgical Management of Massive Oesophageal Hiatal Hernia in Squirrel Monkeys (Saimiri sciureus)
Source: Vet Med Sci. 2025 May 19;11(3):e70404. doi: 10.1002/vms3.70404 (PMC12087505; doi:10.1002/vms3.70404)
Supplement: Supplementary file 1 — Supporting information [file VMS3-11-e70404-s001.docx]

**Supplementary Video 1:** This video presents the early postoperative course following surgical correction of the giant hiatal hernia in the first squirrel monkey. It shows the course of recovery of the monkey with restitution of respiration, return of motility, and early restitution of dietary intake. Key aspects of these observations include behavioral follow-up post-surgery and observation by the clinical team that the animal was stable in this most critical recovery phase.

Supplementary Video 2:

This video documents the post-surgery recovery of the second squirrel monkey concerning extended hospitalization and adjustments in diet. The video describes the gradual acclimatization of the monkey to a changed diet, increased physical movement, and treatment by the veterinary staff aimed at controlling temporary adverse postoperative manifestations such as bloating and indigestion. Relatively better stabilization at discharge underscores the importance of postoperative care for ensuring long-term recovery.
